# Shifting the Level of Selection in Science

**DOI:** 10.1177/17456916231182568

**Published:** 2023-08-01

**Authors:** Leo Tiokhin, Karthik Panchanathan, Paul E. Smaldino, Daniël Lakens

**Affiliations:** 1 Human Technology Interaction Group, Eindhoven University of Technology, The Netherlands; 2 Data & Analytics Group, IG&H, The Netherlands; 3 Department of Anthropology, University of Missouri, USA; 4 Department of Cognitive & Information Sciences, University of California, Merced, USA; 5 Santa Fe Institute, New Mexico, USA

**Keywords:** multilevel-selection theory, cooperation, team science, science policy, recognition and rewards

## Abstract

Criteria for recognizing and rewarding scientists primarily focus on individual contributions. This creates a conflict between what is best for scientists’ careers and what is best for science. In this article, we show how the theory of multilevel selection provides conceptual tools for modifying incentives to better align individual and collective interests. A core principle is the need to account for indirect effects by shifting the level at which selection operates from individuals to the groups in which individuals are embedded. This principle is used in several fields to improve collective outcomes, including animal husbandry, team sports, and professional organizations. Shifting the level of selection has the potential to ameliorate several problems in contemporary science, including accounting for scientists’ diverse contributions to knowledge generation, reducing individual-level competition, and promoting specialization and team science. We discuss the difficulties associated with shifting the level of selection and outline directions for future development in this domain.

The predominant approach to scientific evaluation uses individual-level criteria, such as one’s number of first-authored publications, citations, *h*-indices, journal impact factors, and success in funding acquisition (Carpenter et al., 2014; Ioannidis, 2014; McKiernan et al., 2019; Moher et al., 2018; Morales et al., 2021). This evaluation strategy implicitly assumes that identifying and rewarding the most accomplished individuals is the best way to generate scientific knowledge.

Yet scientists contribute to knowledge production in many ways that are not reflected by individual accomplishments (Moher et al., 2018; Oettl, 2012). Such contributions include being diligent peer reviewers, serving as dedicated mentors, improving the work climate in scientific communities, facilitating communication between fields, engaging in replication research, and detecting fraud and statistical errors in published articles. Scientists can also detract from knowledge production in various ways, including being exploitative mentors, sabotaging competitors, engaging in fraudulent or questionable research practices, and overselling research (Anderson et al., 2007; Chambers, 2017; Ellemers, 2021). Such effects are often not accounted for by evaluation criteria, and even when these effects are considered, they are seen as less important than individual research contributions (Dawson et al., 2022).

Reflecting these concerns, proposals for reform from scholars across disciplines and nations have argued for the need to broaden evaluation criteria (Moher et al., 2018). For example, the Hong Kong Principles for assessing researchers seek to reward behaviors that strengthen research integrity (Moher et al., 2020). Proposals for “responsible indicators for assessing scientists” (RIASs) highlight the need to evaluate contributions including peer review, open and reproducible sharing of data and materials, and communication of research via media outlets (Moher et al., 2018). The Declaration on Research Assessment (DORA) recommends considering the value from all outputs and outcomes generated by research, using a diversity of metrics (DORA, 2012). In the Netherlands, funders and universities have committed to reforming their system of recognition and rewards, moving away from a “one-sided emphasis on research performance” and toward recognizing a wider range of contributions, such as commitment to collaboration, education, and open science practices (NWO, n.d.).

Despite many promising ideas, a major limitation is that reform proposals rely primarily on intuition about which modifications will improve the efficiency and reliability of science. Reform proponents acknowledge this point, noting that “the extent to which these [reforms] can be expected to improve the efficiency and reliability of science remains unknown” (Ioannidis, 2014, p. 5) and that new evaluation criteria “need to be studied in terms of . . . the kind of systems needed to implement them their usefulness in both evaluation and modifying researcher behaviours, and the extent to which each may be gamed” (Moher et al., 2018, p. 11). To move beyond intuition-based reform, a range of scholars have argued that metascience could productively draw on theoretical frameworks from fields with a longer history of addressing related problems (Engel, 2015; Gall et al., 2017; Smaldino, 2019; Tiokhin et al., 2021).

In this article, we illustrate how the theory of multilevel selection from evolutionary biology can provide conceptual tools for structuring scientific reforms and reasoning about their consequences. Multilevel-selection theory is used to analyze situations in which individuals are structured into groups, individual behavior affects others’ outcomes, and competition occurs at different levels of social organization. For example, in team sports, there is both competition between teams (e.g., to win games) and between players within teams (e.g., to get the best contract). Given the presence of such features in academic science, multilevel-selection theory may be particularly relevant for understanding how to modify selection pressures in academia to improve knowledge generation.

The remainder of this article proceeds as follows. First, we outline several problems with the reward structure in academia and explain how these arise from the way that individual researchers are evaluated. Second, we provide empirical examples of how several fields—animal husbandry, team sports, and professional organizations—have addressed this class of problems by shifting the level of selection from individuals to groups. Third, we introduce multilevel-selection theory and associated concepts (explaining how it is possible to shift the level at which selection operates), and provide principles and potential reforms to address problems in the production of scientific knowledge. Fourth, we discuss the difficulties associated with shifting the level of selection in practice and provide directions for future development.

## A Tale of Two Scientists

Imagine two scientists, Kotrina and Amber, who have just obtained their PhDs and are entering the job market in psychology. Kotrina has published two empirical articles. She is first author on one, including a publication in a prominent journal, *Journal of Experimental Psychology: General*. Her articles collectively have 50 citations (with one cited 30 times), she has mentored five undergraduate students, and she has obtained a modest research grant. Amber has published six empirical articles. She is first author on three, including three publications in prominent journals—*Psychological Science*, *Journal of Experimental Psychology: General*, and *Proceedings of the National Academy of Sciences*. Amber’s articles collectively have over 170 citations (with four articles cited more than 40 times each), she has mentored nine undergraduate students, and she has obtained a major research grant.

Imagine that you were a member of a search committee, and Kotrina and Amber were in the running for your department’s final interview spot. Which candidate would you choose?

Given the typical criteria used by departmental selection committees to evaluate scholars (Schimanski & Alperin, 2018), we expect that most committees would choose Amber. After all, Amber has published more articles in more prominent journals and has more citations. Amber has also mentored more students and obtained more funding. A good selection committee may realize that focusing on proxy measures such as publication count, citations, and funding can distort science by incentivizing less rigorous research (Smaldino & McElreath, 2016). Still, relying on standard metrics, it is hard to avoid the conclusion that Amber is doing better work, at a higher rate of productivity, and with more potential for external support. If you needed to select the best individual scientist, Amber would seem like the obvious choice.

### Is choosing the best scientist so simple?

Now, suppose that you talk to colleagues and learn more about each candidate before making a decision. You learn that Amber often acts negligently—she does not carefully document her experimental procedures, check her code for bugs, or make her materials available to others. You also learn that Amber engages in questionable research practices (John et al., 2012) to increase the probability that she obtains statistically significant findings. Consequently, some of Amber’s publications likely contain false-positive findings, which will waste the time of scientists who attempt to build on her work. Amber is so driven to succeed that she neglects many prosocial aspects of being an academic—she rarely performs departmental service or helps colleagues when they ask for assistance, and she writes short and low-quality peer reviews. To top it off, Amber is a terrible mentor. She barely makes time for students, and when she does, colleagues have seen her exploiting students, stealing their ideas without allocating proper credit, and withdrawing mentorship from students who were struggling. Amber is certainly a productive individual, but she is a poor colleague, peer, and community member.

In contrast, you learn that Kotrina typically acts with exceptional diligence—she carefully documents her experimental procedures, double-checks her code for bugs, and makes her materials readily accessible to others. Kotrina works hard to avoid questionable research practices and conducts her research slowly and methodically. As a result, her publications are more likely to contain true findings and make scientific advances, contributing to the gradual accumulation of knowledge. Kotrina is deeply committed to helping people in her community—she serves on departmental committees, assists colleagues whenever they ask for help, and is a thoughtful and constructive peer reviewer. To top it off, Kotrina is a dedicated mentor. Colleagues mention that Kotrina devotes personal time to helping students become better scholars, credits students for their contributions, and steps up her commitment when students are struggling. Kotrina may not be the most productive individual, but she is a wonderful colleague, peer, and community member.

Knowing all of this, would you reconsider your choice? Is it possible to separate Amber and Kotrina’s scientific contributions from their effects on the productivity and well-being of colleagues and the broader scientific community?

### Typical evaluation criteria neglect indirect effects

**Fig. 1.**
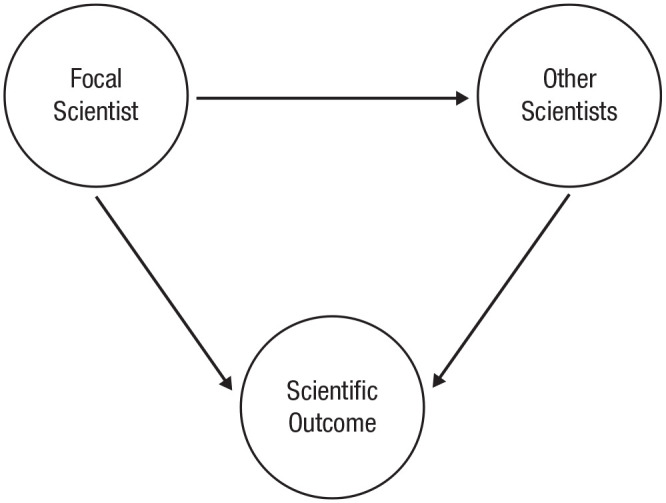
Scientists contribute to any measurable scientific outcome in two ways: directly (focal scientist → scientific outcome) and indirectly (focal scientist → other scientists → scientific outcome). A scientist’s total causal effect on any scientific outcome is the sum of both direct and indirect effects.

The tale of Kotrina and Amber illustrates two pathways by which scientists contribute to science: directly and indirectly. A direct effect is one in which the causal path goes straight from a scientist’s efforts to a measurable scientific outcome. An indirect effect is one in which the causal path from a scientist’s efforts to a measurable scientific outcome goes through other scientists. In other words, indirect contributions are mediated by their effects on other scientists’ direct contributions (see Lucas, 1988, for the analogous idea of internal and external benefits in human capital). For example, scientists can directly affect research production by conducting experiments themselves, and they can do so indirectly by helping colleagues to design and run better experiments. The causal model in Figure 1 illustrates these two pathways.

Every scientist can contribute to science via these two pathways. This means that without accounting for both direct and indirect effects, it is impossible to determine a scientist’s total contribution to any scientific outcome.

And herein lies the problem: many indirect effects are not accounted for in the metrics used to assess scientists’ productivity, such as first-authored publications, *h*-indices, and counts of individual citations. Of course, no metric can capture all relevant factors, so is it really that harmful to rely on criteria that primarily measure direct effects? Are there tangible repercussions for the efficiency of science, the well-being of scientists, the spread of good scientific practices, or other dimensions that truly matter?

### The repercussions of neglecting indirect effects

We see several reasons why neglecting indirect effects has negative repercussions for science. In the short term, neglecting indirect effects fails to reward scientists who help others and fails to penalize scientists who harm others. In the intermediate term, neglecting indirect effects increases competition and reduces cooperation between individual scientists. And in the long term, neglecting indirect effects reduces the incentive for scientists to specialize in roles that are essential for efficient team science.

#### Neglecting indirect effects fails to reward scientists who help others and fails to penalize scientists who harm others

Consider a case in which a scientist generates little direct output, such as first-authored publications. Given current evaluation criteria, such a scientist would struggle to find a research position, obtain grants, and receive awards. Would this be justified?

It depends. If such a scientist generates large positive indirect effects, then their total contribution may be sufficient to warrant recognition and rewards, despite the fact that they produce little output themselves. Many of us are familiar with such individuals—they are not exceptionally productive, but they lift up their departments, improve their colleagues’ productivity, and are a joy to be around.

Next, consider a scientist who generates substantial direct output, including many first-authored publications. Should this scientist be hired, obtain grants, and receive awards?

Again, it depends. If such a scientist also generates large positive indirect effects, then a focus on direct output would lead to an underestimation of their total contributions. If, however, the scientist achieves their productivity at others’ expense, then neglecting indirect effects would lead to an overestimation of their total contribution.

Thus, by neglecting indirect effects, current evaluation criteria fail to adequately reward scholars who benefit science by helping others and fail to adequately penalize scholars who detract from science by harming others.

#### Neglecting indirect effects increases competition and reduces cooperation between individual scientists

Competitive behaviors impose costs on others, whereas cooperative behaviors confer benefits to others (see Table 1 for a taxonomy of social behavior). One consequence of neglecting indirect effects is increased competition and reduced cooperation between scientists. This occurs because indirect effects cause individuals to have a stake in each other’s outcomes, thereby creating a shared fate. In evolutionary biology, it is well established that mechanisms that create a shared fate incentivize individuals to confer benefits to others, reducing individual-level competition and promoting cooperation in many cases (Aktipis et al., 2018; Fletcher & Doebeli, 2009).

**Table 1.** A Taxonomy of Social Behavior

	Effect on others	
Effect on the actor	Positive (cooperative)	Negative (competitive)
Positive	Mutually beneficial	Selfish
Negative	Altruistic	Spiteful

Although competition among individuals can be useful—promoting innovation, increasing effort, and incentivizing scientists to pursue diverse problems (Balietti et al., 2016; Dechenaux et al., 2015; Strevens, 2003)—competition also has costs. Individual-level competition incentivizes scientists to engage in behaviors that benefit themselves, whereas personally beneficial behaviors are only a subset of the behaviors that benefit science. For example, the scientific community plausibly benefits from the open sharing of information such as code, materials, and raw data, whereas individual-level competition disincentivizes information-sharing to hinder competitors’ success (Derex et al., 2014; Mitri et al., 2009). Surveys and focus-group discussions provide evidence that scientists strategically withhold information in competitive contexts to minimize the probability that others find flaws in their work or succeed in the race for priority of discovery (Anderson et al., 2007; Hagstrom, 1974).

Individual-level competition also incentivizes scientists to be indifferent to their effects on others and even to strategically harm others to obtain a competitive advantage. Economic models of public goods demonstrate that without penalties for negative externalities, individuals are more strongly incentivized to engage in selfish behavior that has collectively harmful consequences, such as cutting corners, conducting questionable research practices, or fabricating data (Engel, 2015). Incentives for strategic harm occur when a competitor’s failure improves the actor’s chance of success, such as two labs competing for priority of discovery or two principal investigators competing for the same grant. Such incentives also occur when an individual can increase productivity by exploiting others, such as taking advantage of students. Laboratory experiments of stylized peer-review systems demonstrate that competition indeed causes individuals to review competitors’ work more negatively (Balietti et al., 2016). Focus-group discussions with scientists at major research universities indicate that scientists engage in many harmful behaviors to succeed in competition, including strategically misreporting research findings to sabotage competitors’ progress, delaying peer review of competitors’ papers to “beat them to the punch,” and lying to and exploiting doctoral students to ensure progress on projects (Anderson et al., 2007).

Competition and the pursuit of self-interest can make populations worse off, particularly when individuals have competing interests and when competitive success is achieved by investing in traits that diminish individual welfare (R. H. Frank, 2012). In academia, selecting scientists on the basis of individual productivity while neglecting indirect effects generates intense individual-level competition, exacerbating the disconnect between the behaviors that lead to successful scientific careers and those that improve the quality of the scientific literature and the well-being of scientists (Munafò et al., 2017; Nosek et al., 2012; Smaldino & McElreath, 2016).

#### Neglecting indirect effects reduces the incentive to specialize in roles that are essential for efficient team science

The widespread reliance on metrics that target direct individual contributions creates an additional problem: Scientists have few incentives to specialize in roles in which they primarily assist others, even though such roles are essential for efficient team science. Team science benefits from specialists whose primary role is to generate positive indirect effects for other team members (e.g., dedicated statisticians, programmers, or facilitators of communication; Forscher et al., 2023; Wuchty et al., 2007). More generally, groups benefit from diverse sets of specialists because individuals who specialize can achieve mastery that surpasses that of generalists: teams of specialists with complementary skills regularly outperform teams of generalists with overlapping skills, even if the generalists are top performers (Page, 2008). Over time, selection for efficient groups leads to cooperative entities that are more than the sum of their individual parts. Such entities involve efficient divisions of labor, systems of communication to coordinate cooperation, mechanisms to suppress competition, and entities that have a shared fate and can no longer function independently (e.g., genes to genomes, cells to multicellular organisms, multicellular organisms to colonies; Szathmáry & Smith, 1995; West et al., 2015).

In contrast, focusing on direct individual contributions pushes scientists away from roles that primarily help their team members and toward roles that receive individual recognition, such as being a team leader or principal investigator. This leads to the situation lamented by Kurt Vonnegut, in which “everyone wants to build and nobody wants to do maintenance” (Vonnegut, 1997, p. 167).

## Lessons From Animal Husbandry, Team Sports, and Professional Organizations

How can indirect effects be accounted for to better align the interests of individual scientists with broader scientific goals? To address this question, we draw on insights from three fields—animal husbandry, team sports, and professional organizations—that have long dealt with similar challenges. Each of these fields has recognized the need to move away from individual-level evaluation to improve group-level outcomes, and all of them have used a shared principle for doing so: shifting the level of selection away from individuals and toward the larger groups in which individuals are embedded.

### Animal husbandry

Farmers aim to implement a breeding strategy that maximizes profits. In poultry, this amounts to maximizing hens’ lifetime egg production. One sensible approach might be to select the most productive individual chickens to reproduce. The reality, however, is not so simple. The causal pathways that affect individual hens’ egg production are complicated because each hen’s productivity is influenced by the behavior of other hens in their social environment. It turns out that the most productive hens in a coop are also the nastiest hens, feather-pecking and cannibalizing the other hens in their coop. Because individual hens who are most productive are those that harm others, selectively breeding the most productive hens can actually lead to lower overall egg production (Muir, 2005; Wade et al., 2010).

From an economic perspective, feather-pecking and cannibalism are problems when they cut into profits (El-Lethey et al., 2000). From an animal-welfare perspective, they are tragedies. Yet breeders have developed a strategy to address these problems: Instead of selecting the most productive individuals, breeders can select the most productive groups—that is, all hens in the most productive coops are selected to reproduce. Such a selection regime implicitly accounts for hens’ indirect effects on group members (Wade et al., 2010). In one application of this approach, mortality dropped from 68% to 9% in just a few generations, and laying increased from 91 to 237 eggs (Muir, 1996).

### Team sports

Sports managers want their teams to win. To accomplish this goal, managers must evaluate and preferentially select players who make the largest positive impact on team performance. Selecting players with impressive individual performance metrics, such as goals scored, may seem like the obvious approach. However, as with chickens who lay many eggs, players with impressive individual metrics are not always the ones who have the most positive impact on their teams. For instance, soccer players who always shoot and never pass increase their chances of scoring goals but reduce their team’s probability of winning games relative to players who pass when others are in a better position to score. Thus, evaluations of professional athletes in team sports rely not only on metrics of individual performance, but also on metrics that capture indirect effects (Berri & Bradbury, 2010; Duch et al., 2010).

In the National Hockey League, the “plus-minus” statistic provides information about a team’s performance when a player is both on and off the ice, thereby helping coaches to decide which players put their teams in position to win. Other statistics measure goal-scoring attempts, shot quality, shots blocked, and whether success was due to luck, and they also attempt to control for context (such as garbage-time play or differences between rinks; Nandakumar & Jensen, 2019).

In Major League Baseball, managers and fans once relied on individual statistics, such as batting average and home runs. However, such metrics did not measure many indirect effects and were poor predictors of team wins. Currently, managers use metrics that capture both direct individual performance (such as slugging percentage and weighted on-base percentage) and indirect contributions to team performance (such as “plus-minus,” weighted runs created, defensive runs saved, and value over replacement player; Beneventano et al., 2012). It is also recognized that many contributions (such as leadership and improving team morale) are difficult to measure with metrics because of the complicated causal chains between players’ actions and team performance (Silver, 2012).

### Professional organizations

Like sports teams, organizations seek to hire and invest in employees who improve organizational productivity. One approach is to hire “stars,” individuals who are exceptionally productive and innovative (Ernst et al., 2000; Groysberg et al., 2008). Although such an approach works in some cases, a focus on productive individuals can also harm organizational outcomes. Again, the problem arises because metrics of individual productivity rarely capture the indirect ways that individuals impact organizations (DeLong & Vijayaraghavan, 2003; Housman & Minor, 2015; Pentland, 2012). For example, some employees are “charismatic connectors” who facilitate communication among team members (Pentland, 2012). Others score high on measures of network centrality, having large, dense, or far-reaching networks of collaborators (Grigoriou & Rothaermel, 2014). Although such individuals may not be individually productive, they can help an organization to achieve its goals by creating more productive interactions among team members or by promoting innovation by recombining knowledge from disparate sources.

Organizations that overemphasize the direct contributions of stars also run the risk of overlooking the ways in which stars indirectly harm organizations. Stars can constrain the emergence of new leaders, both because organizations allocate disproportional resources to support the star’s research program and because stars have incentives to prevent other employees from advancing to high-status roles (Kehoe & Tzabbar, 2015). Stars can dominate discussions and champion their own ideas, whereas better solutions would have arisen had a diverse set of individuals contributed in a more democratic system (Page, 2008; Woolley et al., 2010).

A further problem is that of star employees who are individually productive but harm fellow employees and corrupt the organizational culture (“toxic workers”). Toxic workers are selfish and overconfident; they engage in harassment, funnel organizational resources toward personal goals, and act unethically in various other ways. One analysis of productivity among workers at a company that built and deployed job-testing software estimated that removing productive-but-toxic workers would increase firm profits even more than hiring star employees (Housman & Minor, 2015).

To mitigate such problems, organizations make use of group-level incentives (Suff et al., 2008). In the simplest form, organizations place employees into teams with shared goals (Hansen, 1997). Many organizations also create explicit incentive schemes in which employee payoffs are tied to the success of the organization, such as profit sharing (where salaries depend on company profits). Some organizations also provide employees with stock options or allow employees to buy company stock at discounted prices. Another approach is to offer team-based performance incentives, in which employees are rewarded when their team meets specific standards or output targets (increasing sales, increasing efficiency, or successfully accomplishing a project). Such group-level incentives encourage employees to identify more with their organization, aligning the interests of individuals and the larger group in which they are embedded (Pendleton et al., 1998).

## Multilevel-Selection Theory

Animal husbandry, team sports, and professional organizations illustrate an overarching lesson. If the goal is to foster group productivity, then it is essential to account for the indirect effects of individuals’ behaviors on other group members by shifting the level of selection from individuals to groups. Shifting selection to the level of groups creates incentives for within-group cooperation and reduces incentives for within-group competition. This occurs because group-level selection creates a shared fate among group members, wherein each individual’s success becomes tied to the success of the group. Shifting the level of selection from individuals to groups favors the spread of behaviors, norms, and institutions that promote group success, regardless of whether individuals consciously understand the underlying causal processes (Boyd & Richerson, 2002; Derex et al., 2019).

More formally, cooperation and competition are examples of what biologists call *social behaviors*—behaviors that have fitness consequences for both the actor and other individuals (West et al. 2007). Social behaviors can have either positive or negative effects on an actor and other individuals (Table 1). We label behaviors in which actors confer benefits on others as “cooperative” and behaviors in which actors impose costs on others as “competitive.” Among the competitive behaviors, “selfish” behaviors benefit the actor, and “spiteful” behaviors harm the actor. Among the cooperative behaviors, “mutually beneficial” behaviors benefit the actor and “altruistic” behaviors harm the actor.

Biologists use multilevel-selection theory to study systems that are hierarchically organized (e.g., genes grouped into cells, cells grouped into individual organisms, individual organisms grouped into groups) in such a way that evolution can simultaneously operate at multiple hierarchical levels (Okasha, 2006; Wilson, 1975; also see Gardner, 2015). A key insight is that strong selection between groups can favor the evolution of cooperation and suppression of competition within groups, because cooperative groups can outcompete selfish groups.

Two factors determine the dominant level at which selection operates: the relative intensity of competition within versus between groups and the extent of variation in social behaviors within and between groups (Okasha, 2006). Of course, biological and cultural evolution are not identical, and researchers in the field of cultural evolution have spent decades exploring the origins, taxonomies, and evolutionary consequences of cultural transmission (Boyd & Richerson, 1988; J. Kendal et al., 2011; R. L. Kendal et al., 2018). There are many specific mechanisms by which group-level selection can occur among human cultural groups (e.g., warfare, group extinction, imitation across groups, selective migration to higher-functioning groups), and formal models have demonstrated the plausibility of these mechanisms (Boyd & Richerson, 2002; Henrich, 2004; Richerson et al., 2016).

When individuals fiercely compete with members of their own group, they are incentivized to act selfishly and disincentivized from acting altruistically, as success becomes a zero-sum game. However, when competition is weaker within groups and stronger between groups, selection at the group level dominates. Individuals then have fewer incentives to engage in selfish behavior because personal success does not depend as much on outcompeting fellow group members. At the extreme, when there is no competition within groups, the only way that individuals can improve their personal success is by improving the success of their group (S. A. Frank, 2003). High between-group competition thus creates a situation of shared fate: Every individual’s success becomes dependent on the group’s success in intergroup competition, incentivizing less competition and more cooperation with fellow group members.

The above insights have been formalized mathematically in a framework known as the Price equation (named for its creator, George Price; for additional information and derivation, see Kerr & Godfrey-Smith, 2002; Okasha, 2006). The Price equation is a general model of evolutionary change that applies to any mode of information transmission (McElreath & Boyd, 2008; for the difference between general and specific models, see Parker & Smith, 1990).

To represent evolution at multiple hierarchical levels, the Price equation is written as

(1) $$\bar{w}\Delta \bar{z}=\mathrm{Var}(z_{g})\beta (w_{g},z_{g})+E[\mathrm{Var}(z_{ig})\beta (w_{ig},z_{ig})]$$

The evolving trait (e.g., a scientist’s level of altruism) is denoted by *z*. Fitness is denoted by *w*. Bars above letters denote average values in the population; Δ denotes the change in the average value of *z* in one generation. The *g* and *i* subscripts index different groups and different individuals within groups, respectively. Var(*z_g_*) represents the variation in the trait between groups and Var(*z_ig_*) represents the variation in the trait within a group. β(*w_g_,z_g_*) represents the regression of group fitness on the trait value of the group, and β(*w_ig_, z_ig_*) represents the regression of individual fitness on the trait value of the individual (Panchanathan, 2011).

To increase the strength of selection for a behavior in a population (e.g., more group-beneficial behaviors, fewer cutthroat individualists), it is necessary for the left-hand side of the equation—the change in average trait value in a population—to be positive. In the case of altruism, altruistic behavior is individually costly but beneficial to others—formally defined as β(*w_ig_,z_ig_*) < 0 and β(*w_g_,z_g_*) > 0. Altruistic traits can increase in frequency in the population when between-group selection for altruism is sufficiently strong to overcome the within-group disadvantage faced by altruistic individuals.

## Shifting the Level of Selection in Practice

In this section, we provide an overview of reforms that could be used to shift the level of selection in science. In practice, shifting the level of selection will require dealing with substantial challenges, including identifying the relevant level of hierarchical organization (ranging from research labs to scientific fields to countries), desirable outcomes to incentivize (such as theoretical progress or solutions to societal problems), the mechanism by which selection is implemented (such as grant allocation or criteria for hiring and promotion), and a range of additional complications (see Limitations). Given the early stages of our understanding of how group structure affects the evolution of scientific practices, there does not yet exist sufficient evidence-readiness to strongly advocate for specific policies (IJzerman et al., 2020). We hope that this section inspires further discussion about how principles from multilevel-selection theory could be used to shape scientific practice and evaluate the consequences of existing proposals for reform.

One subset of reforms could shift scientific evaluation toward more strongly considering group outcomes. Just as companies provide employees with stock options and bonuses based on company performance, promotion or rewards could be made partially contingent on the performance of groups in which scientists are embedded (e.g., departments). Other reforms could fund permanent positions for individuals whose primary role is not to produce research but rather to help others improve their research output. Such positions could include departmental statisticians, technicians, data managers, mentors and instructors, and individuals who facilitate communication among scientists (Teperek et al., 2022).

Other approaches might develop ways to more formally account for indirect effects. Given current unsystematic approaches to evaluating indirect effects (e.g., using letters of recommendation to assess collegiality, weighing contributions to articles based on authorship order), there is substantial room for innovation in this area. Progress has already been made in certain domains, such as the development of the Contributor Roles Taxonomy (CRediT; McNutt et al., 2018) and citation-based algorithms (Shen & Barabasi, 2014) to determine collective credit allocation in multiauthor articles. Metrics of network centrality may be useful for determining which scientists have large, dense, or far-reaching networks of collaborators or have the potential to fill structural holes (Burt, 2004; Li et al., 2013), an approach that has shown promise in other fields (Duch et al., 2010). Narrative curriculum vitaes (CVs)—which allow candidates to describe a wider range of contributions than traditional ones—have been adopted by some funders and hold promise for revealing a wider range of indirect effects (Singh Chawla, 2022). However, it is unlikely that narrative CVs will capture ways in which scientists harm scientific progress (e.g., generating unreliable results by engaging in questionable research practices). This points to the need to develop better metrics to capture the harmful effects of scientists’ behaviors.

Additional reforms to shift the level of selection could create competitions that incentivize larger-scale cooperation. Corporations such as Netflix have successfully incentivized group-level competition by offering financial prizes for improvements to their movie-recommendation algorithm (“Netflix Prize,” 2021). Science funders can create competitions to funnel scientists’ efforts toward critical outstanding problems. Such an approach is already used by the Clay Mathematics Institute, which offers 1 million USD for correct solutions to unsolved problems in mathematics (“Millennium Prize Problems,” 2021). Funders can also encourage antagonistic collaborations between competing teams, with the goal of reconciling conflicting findings and developing theoretical consensus. Such an approach was recently implemented by the Templeton Foundation to fund structured adversarial collaborations to test competing theories in the research area of consciousness (Accelerating Research on Consciousness, n.d.).

Group-level competition can be fostered by facilitating interaction between groups that would otherwise remain isolated, such as scientific fields. For example, Smaldino and O’Connor (2020) built a specific model of the evolution of scientific methods in a community-structured population. Their model demonstrated how community structure—formalized as the assignment of credit and the sharing of methods between communities—can help overcome community-specific shortcomings that would otherwise stymie the spread of superior methods. Such findings highlight the importance of complementing initiatives to promote field-level diversity with opportunities for interfield contact and competition.

Other reforms could suppress competition within groups, as selection is shifted upward by mechanisms that reduce within-group competition (S. A. Frank, 2003), thereby increasing the relative variation between groups. One possibility is to develop institutions that produce more equal outcomes, such as departments in which individual faculty members’ resources are partially redistributed among fellow faculty or in which there are limits on the number of graduate students that can work with a principal investigator.

Another leveling mechanism is randomization. Randomization places individuals behind a “veil of ignorance” about their future outcomes (Rawls, 1971) so that each individual can only increase their chance of success by increasing the average success of the group. As with redistribution, randomization reduces inequality in expected outcomes within groups. Unlike redistribution, randomization acts like a lottery: In any instance, one individual receives a disproportionately large payoff. Consequently, randomization is preferable in situations of increasing marginal returns, whereas redistribution is preferable in situations of diminishing marginal returns (Nettle et al., 2011). In the context of grant funding, formal models demonstrate that randomization can increase scientific efficiency and decrease the individual-level competition that selects for reduced rigor (Gross & Bergstrom, 2019; Smaldino et al., 2019).

Another way to suppress competition is to make it easier for individuals to police group-detrimental behaviors. One approach could be to fund independent entities to evaluate research quality or conduct audits of researchers (Barnett et al., 2018). Examples include the National Academy of Sciences’ Strategic Council for Research Excellence, Integrity, and Trust in the United States (McNutt et al., 2021) and random audits of research methods, procedures for collecting data, and misconduct reporting (Tilburg University Science Committee, n.d.; Van Noorden, 2014). Other possibilities include providing grants and permanent positions for individuals who engage in scientific criticism, ranging from rigorous peer review to fraud detection to “red teams” of independent critics (Lakens, 2020; Vazire & Holcombe, 2020).

### Limitations

We have emphasized the analogical similarities between academic science and several disparate fields. By mapping new target domains onto previously encountered base domains, analogies have provided a key tool for generating scientific discoveries (Klahr & Simon, 1999). However, any single analogy provides just one lens through which a problem can be viewed. In this section, we provide an overview of potential limitations and concerns regarding shifting the level of selection in science.

One concern is the possibility of gaming. Campbell’s Law states that “the more any quantitative social indicator is used for social decision-making, the more subject it will be to corruption pressures and the more apt it will be to distort and corrupt the social processes it is intended to monitor” (Campbell, 1979). Gaming is a problem regardless of the level at which selection operates and is an issue in many sectors (Larkin, 2014). However, gaming is particularly worrisome in instances when there is a weak link between the target of selection (e.g., number of citations) and the desired outcome (e.g., theoretical progress).

Although we have focused on the benefits of large-scale cooperation, not all science will benefit from such an approach. Similarly, not every scientist will make a larger contribution by engaging in more collaborative research. Although some tasks are insurmountable without large-scale cooperative teams—CERN’s efforts to build the Large Hadron Collider, for example, involved collaboration among over 10,000 scientists across 100 countries—other tasks benefit from smaller-scale efforts. A recent large-scale analysis of patents, software projects, and academic publications between 1954 and 2014 found that large research teams tended to develop existing ideas, whereas small teams were more likely to generate disruptive ideas that constituted scientific and technological breakthroughs (Wu et al., 2019). Science policies must find ways to promote larger-scale cooperation while also valuing smaller-scale research and rewarding a diversity of team sizes.

Additional work is needed to determine the optimal strategy for weighing individual versus group contributions as well as to establish the conditions in which shifting selection upward will benefit science and improve welfare. Larger-scale cooperative groups can more effectively harm competing groups (Zefferman & Mathew, 2015). Further, although group-level selection can improve welfare, it may also reduce it (e.g., despotic political regimes rising to power by promoting institutions that allow them to dominate and assimilate more democratic neighboring polities; Turchin, 2016). Reforms to shift selection upward should thus be supplemented by regulations to prevent welfare-reducing outcomes. For example, in professional hockey, league-level mandates for helmets were necessary to ensure players’ safety, as any players did not want to wear helmets for fear of losing their competitive edge (R. H. Frank, 2012).

Another issue is the potential to crowd out moral incentives with external rewards for performance (Bowles, 2016). Much of science is characterized by group-beneficial behaviors that generate no financial rewards but are undertaken because of reputational concerns, reciprocal relationships with colleagues, or embodiment of prosocial norms (Merton, 1973). Such behaviors include peer review, providing feedback on colleagues’ manuscripts, mentoring students, writing letters of recommendation, and giving talks. Because adding external rewards can reduce the weight placed on internal rewards, this can result in even lower levels of the rewarded behavior (Gneezy & Rustichini, 2000). The extent of crowding out is difficult to anticipate, and lab experiments and smaller-scale pilot interventions are key tools for understanding its potential consequences.

A final concern is the possibility that policy interventions change the dynamics of a system in unforeseen ways, leading to unintended consequences. This concern applies to any intervention in any complex system, including shifting the level of selection in science and open science reforms more broadly (Field, 2022). Potential strategies for dealing with this problem include using crowdsourcing methods (Lenart-Gansiniec et al., 2022) to anticipate a wider range of outcomes, developing aggregate health indicators to track the pre- and postintervention health of scientific systems, and using system-dynamics simulations to reveal unrecognized connections within complex system interventions (Stephens & Atwater, 2019).

### Conclusion

Scientists are primarily evaluated on the basis of their individual performance. Such evaluation criteria create a disconnect between what is best for scientists’ careers and what is best for science. Problems generated by a focus on direct individual contributions include failing to account for the indirect ways that scientists contribute to science, incentivizing selfishness and disincentivizing cooperation, intensifying competition between scientists, and hindering specialization and the emergence of large-scale cooperative teams. Multilevel-selection theory provides a framework for understanding how to address these problems and has been productively applied in several fields to improve group outcomes. The key principle is to account for indirect effects by shifting the level of selection away from the level of individuals and toward the level of groups. Shifting selection to the group level creates a shared fate among group members, fostering cooperation and hindering competition within groups. Shifting the level of selection is far from simple, and its practical application requires further study. Yet, given its theoretical promise and empirical track record, shifting the level of selection should be considered as an overarching principle for structuring scientific reform.

## Transparency

*Action Editor:* David A. Sbarra

*Editor:* Interim Editorial Panel

*Author Contributions*

All authors benefit both individually and collectively from publishing and disseminating this article. L. Tiokhin developed the idea for the article and was responsible for the final structure. All authors contributed to conception, drafting, and revision, and all agree to be accountable for the work.
